# Cost of Treatment for Cervical Cancer in India

**DOI:** 10.31557/APJCP.2020.21.9.2639

**Published:** 2020-09

**Authors:** Maninder Pal Singh, Akashdeep Singh Chauhan, Bhavana Rai, Sushmita Ghoshal, Shankar Prinja

**Affiliations:** 1 *Department of Community Medicine and School of Public Health, Post Graduate Institute of Medical Education and Research, Chandigarh, India. *; 2 *Department of Radiotherapy, Post Graduate Institute of Medical Education and Research, Chandigarh, India.*

**Keywords:** Cervical cancer, treatment cost, out of pocket expenditure, health system costing, health benefit package

## Abstract

**Introduction:**

Cervical cancer is a major public health problem in India leading to high economic burden, which is disproportionately borne by the patients as out-of-pocket expenditure (OOPE). Several publicly financed health insurance schemes (PFHIs) in India cover the treatment for cervical cancer. However, the provider payment rates for health benefit packages (HBP) under these PFHIs are not based on scientific evidence. We undertook this study to estimate the cost of services provided for treatment of cervical cancer and cost of the package of care for cervical cancer in India.

**Methods:**

The study was undertaken at a large public tertiary hospital in North India. The health system cost was assessed using a mixed micro-costing approach. The data were collected for all the resources utilized during service delivery for cervical cancer patients. To evaluate the OOPE, randomly selected 248 patients were interviewed following the cost of illness approach. Logistic regression was used to assess the factors associated with catastrophic health expenditure (CHE).

**Results:**

Health system cost for different cervical cancer treatment modalities i.e. radiotherapy, brachytherapy, chemotherapy and surgery, ranges from INR 19,494 to 41,388 (USD 291 – 617). Furthermore, patients spent INR 4,042 to 23,453 ( USD 60 – 350) as OOPE. Nearly 62% patients incurred CHE, and 30% reported distress financing. The odds of CHE (OR: 25.39, p-value: <0.001) and distress financing (OR: 15.37, p-value: 0.001) were significantly higher in poorest-income quintile. The HBP cost varies from INR 45,364 to 64,422 (USD 676 – 960) for brachytherapy and radiotherapy respectively.

**Conclusion:**

Cervical cancer treatment leads to high OOPE in India, which imposes financial hardship, especially for the poorest. The coverage of risk pooling mechanisms like PHFIs should be enhanced. The findings of our study should be used to set the reimbursement rates of providing cervical cancer treatment under PFHI schemes.

## Introduction

Globally, cancer is one of the major causes of deaths, contributing to 8 million deaths every year (Ferlay et al., 2015). Cervical cancer has become a major public health problem worldwide as its second most common cancer among women, and fourth most common cancer overall. It is also the fourth leading cause of mortality among women worldwide. Countries which are low in development status (Human Development Index; HDI<0.80) account for about 84% cervical cancer cases and 88% of the deaths due to cervical cancer. India accounts for highest absolute number of deaths attributed to cervical cancer (Arbyn et al., 2020). In terms of cervical cancer cases, India represents one-fourth of its global burden and 70% of the burden in the South East Asia Region (Bray et al., 2013). Further, cervical cancer accounts for 17% of all cancer deaths among women of age group 30-69 years in India (Bobdey et al., 2016).

For most of the cancer patients in India, the cost of treatment is borne as an out-of-pocket expenditure (OOPE) (Rajpal et al., 2018). This puts an exorbitant strain on household finances. In India, various publicly financed health insurance schemes (PFHIs) have been implemented by the centre and state governments for financial protection covering cervical cancer treatment (Prinja et al., 2017a). One of the problems in effective planning for such schemes is limited availability of published data on the cost of cancer treatment. In such situation, the reimbursement prices of most of the health benefit packages (HBPs) under PFHIs are based on a consultative process with experts and not on any scientifically driven cost information.

We could find a single study which estimated the total cost of head and neck cancer treatment from a societal perspective (Chauhan et al., 2018). Although, there are few economic analysis of cancer treatment, most of these have only assessed OOPE only (Mahal et al., 2013; Mohanti et al., 2011; Mondal et al., 2014; Rajpal et al., 2018). Moreover, as India has introduced the world’s largest government-funded healthcare insurance programme – Ayushman Bharat Pradhan Mantri-Jan Arogya Yojana (AB PM-JAY), there is an urgent need of generating cost estimates for establishing provider payment prices of HBPs (Angell et al., 2019; Prinja et al., 2020a). It is very important to provide treatment of cervical cancer which is accessible and affordable to the Indian population. Considering this background, the present study was designed for estimating cost of the HBPs (inclusive of both the health system cost and OOPE) of various treatment modalities for cervical cancer treatment. Such evidence is critical to design effective policy interventions in India to provide free universal care for cervical cancer treatment in public hospitals and beneficiaries of PFHIs. 

## Materials and Methods

*Study setting*

The present study was conducted in the Departments of obstetrics and gynaecology (OBG) and radiotherapy of a tertiary care public sector hospital located in North India. The hospital has facilities for provision of surgical care, radiotherapy, brachytherapy and chemotherapy for cervical cancer treatment. 

*Treatment process*

Any patient with symptoms of cervical cancer first reports to the outpatient clinic (OP) of the OBG department. After initial investigations (like biopsy, blood tests, etc.) and clinical examination, the diagnosis is confirmed. The treatment plan for each patient is decided at this level, where surgical treatment is offered if required. For further management i.e. radiotherapy, brachytherapy and chemotherapy, the patient is referred to the department of radiation oncology. 

*Data collection*

*Health system cost*

The health system cost i.e. cost incurred to the hospital, was assessed following the concept of economic costing and mixed (top-down and bottom-up) micro-costing approach (Chapko et al., 2009; Drummond et al., 2005; Prinja et al., 2014). Using this methodology, the first step involved identification and classification of cost centres (direct and indirect). The direct cost centres involved in cancer treatment were the outpatient clinic (OP), inpatient ward (IP), radiotherapy units etc. Similarly, supportive or indirect cost centres like laboratory, dietetics, laundry, etc. were identified. In the second step, data on the quantity of various inputs i.e., both capital and recurrent resources spent on the delivery of services was collected for the reference year of 2016-17. 

To assess the building space used to provide patient care facility maps were obtained from the engineering department. This is a standard methods to obtain the dimensions of different rooms, corridors, operation theatres, procedure rooms etc (Prinja et al., 2020b). Facility maps contain detailed specification of the dimensions of each room within the building prepared by the planning division of hospital engineering. This was used to determine the total floor area of space which was used in the rooms designated to deliver various services for treatment of cervical cancer patients. In addition, the area of the common spaces used for services provided to patients of cervical cancer and other disease conditions, as well as non-patient care services such as waiting area was also elicited from the maps. Further, the non-consumable stock registers were reviewed for assessing the quantity of equipment and furniture items used in different cost centres for provision of care. Similarly, recurrent resources (drugs, consumables, surgical supplies, sanitary and stationary items) were estimated by reviewing the stock registers, indents, vouchers and pharmacy records. Data on the salaries received by all the staff members involved partly or completely in the cancer treatment was assessed from the accounts department. The number of diagnostic tests prescribed to the patients were assessed from the admission files. In the third step, data on the output produced by each of the cost centres (number of out-patient consultations, in-patient admissions, surgeries, radiotherapy sessions, etc.) were assessed from the annual report and department records. 

The final step was assigning a monetary value to each of the inputs. For estimating space costs, the current market rental price of a similar space was assessed based on key informant interviews. The procurement prices of equipment, drugs and consumables, were obtained from the procurement department and central store of the study hospital. For the furniture items, where procurement prices were not available, market prices were used. The cost of overheads like electricity, water, maintenance, laundry and dietetics was obtained at the hospital level. Further, data on actual consumption of expenditure on electricity in each of the rooms was obtained based on the assessment by the electrical engineers of the hospital. The cost of various diagnostic tests, were used as reported in a recent study conducted in the same hospital (Sangwan et al., 2017).

Both the medical and the technical staff members involved in patient care were interviewed for assessing their time spent on various activities. The medical staff were interviewed for time spent on activities carried out on regular basis (operation theatre, radiotherapy treatment etc.) and fixed-interval (meetings, teaching etc.) i.e. weekly, monthly, annually etc. Similarly, technical staff related to radiotherapy treatment were interviewed for time spent on planning (like CT simulation, contouring, dosimetry etc.), quality assurance and radiotherapy delivery. Alongside these interviews, observational data were collected to calculate per patient time spent on CT simulation, planning and radiotherapy delivery. A total of 3 consultants, 4 senior and junior residents each, a medical physicist and 3 technical staff members were interviewed and subsequently observed. 

*Out of Pocket expenditure*

“Cost of Illness” approach, which classifies OOPE into direct and indirect health-care expenditure was used (Rice, 2000). Direct health expenditure included expenses incurred on the user fee, diagnostic tests, drugs, consumables etc. Further, the direct non-health expenditure included expenses on transportation, boarding/lodging and food for patient as well as the caregiver. As the main aim of the study was to estimate the cost of cancer treatment to inform price-setting, the indirect health care expenditure incurred by the households were not estimated. 

Data on OOPE was elicited from 2 groups of patients (n=248). The first group comprised of patients (n=64) who were recruited at the time of registration in the department of radiotherapy and were prospectively followed-up for the entire duration of treatment. The second group consisted of patients (n=184) who had completed their treatment (within the last 6 months) and were retrospectively interviewed at the time of their follow-up visit. For the first set of patients, all new registrations during the period of data collection were approached on a daily basis for recruitment in the study. For the second set, all post-operative cases, visiting the OP for follow up were asked for willingness to participate. 

The recruited patients were interviewed based on a pre-tested semi-structured interview schedule, adapted from previous studies done in the similar settings (Chauhan et al., 2018; Prinja et al., 2019; Prinja et al., 2018; Prinja et al., 2017b; Prinja et al., 2016). It included information on socio-demographic characteristics, duration of treatment, consumption expenditure, insurance status, OOPE incurred on diagnosis/treatment and coping mechanisms for dealing with the same. Payment receipts and bills were checked where available to validate the reported expenditure. Expenditure incurred on pre-radiotherapy treatment (in the gynaecology department) and specifically on surgery (if any), was elicited retrospectively from both the groups. If the patient had taken any treatment before coming to the study hospital, OOPE on account of the same was also recorded. 

*Data analysis*

*Health system cost*

Capital costs were annualized to arrive at the equivalent annual cost taking into consideration a 3% discount rate and the lifespan of the capital equipment (Baltussen et al., 2003; Drummond et al., 2005). The average life of the equipment was determined based on the interview with the staff members involved in using these equipment. 

Space cost was calculated by multiplying the estimates of floor size of the facility with the local commercial rental price to estimate the opportunity cost of space. The total cost of the recurrent resources (drugs and consumables) was estimated by multiplying the unit price with the quantity of respective resource consumed. Certain resources (both capital and recurrent) were used to deliver 2 or more services, and hence classified as joint costs. Such costs were apportioned to respective services using appropriate apportioning statistics. The proportional time spent by staff members in various activities was used as an apportioning statistic for allocating their salaries to each of the respective activity. 

*Out -of-pocket expenditure (OOPE)*

Mean OOPE (with 95% confidence interval) incurred on specific therapeutic modality i.e., surgery, radiotherapy, brachytherapy and chemotherapy was estimated. Its distribution in terms of direct-health and direct non-health care expenditure was also assessed. The extent of financial risk protection was measured in terms of the prevalence of catastrophic health expenditure (CHE) and distress financing. Expenditure on cancer treatment which exceeded the threshold of 40% of non-food household consumption expenditure was considered as CHE (Moreno-Serra et al., 2011; World Health Organization, 2015). Those patients who reported borrowing (with or without interest) or selling of assets (land, jewellery, etc.) to cope the health care expenditure were categorised as facing distress financing (Huffman et al.,2011; Prinja et al.,2016). All the cost and expenditure estimates in the present study pertain to the year 2016-17. Sensitivity analysis was carried out to assess the change in the prevalence of CHE by the varying the cut off from 20% to 50%.

*Health Benefit Package (HBP)*

The HBP cost includes health system cost and OOPE. The health system cost of HBP is sum of unit cost data for all the individual services within an HBP i.e. OP consultation, diagnostics inpatient/intensive care, surgery/radiotherapy/brachytherapy etc. The unit cost per service was multiplied with the frequency of each service utilised during the treatment.

*Ethical consideration*

Ethical approval was obtained from the Institute Ethics Committee, Post Graduate Institute of Medical Education and Research, Chandigarh, India (Reference number: IEC-12/2017-786). Written informed consent was obtained to interview the patients and staff members. 

## Results

*Health system cost*

*Unit cost*

The unit cost per outpatient consultation in the department of OBG and radiotherapy was estimated to be INR 324 and INR 547 respectively. Further, per bed day cost of hospitalization was found to be INR 2,742. Specifically, the health system cost incurred on various treatment options varied from INR 19,494 to INR 41,388 for a patient treated with radical hysterectomy and 3-dimensional radiotherapy respectively (Table 1).

*Input wise distribution of cost*

**Figure 1:**
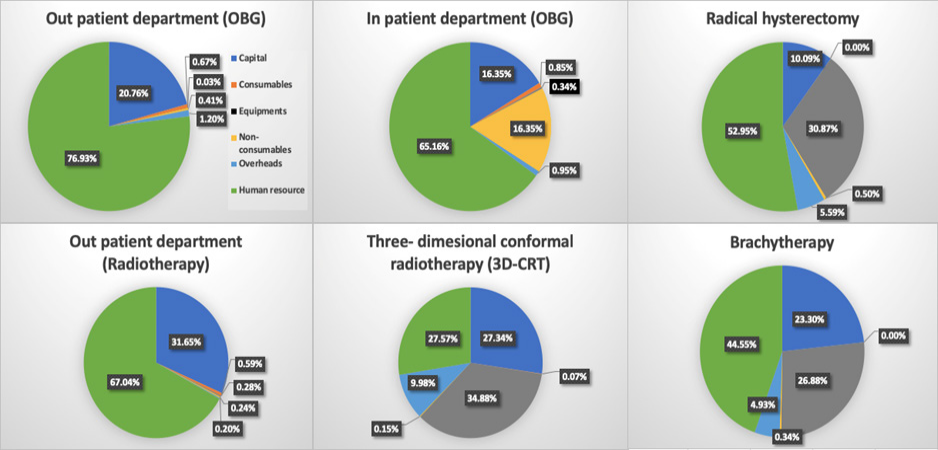
Input-Wise Distribution of Annual Health System Cost for Various Services Delivered for Cervical Cancer Care

The detailed input wise break up of cost incurred on different services related to cervical cancer care is shown in figure 1. More than two-thirds of the total cost (77% and 65%) incurred on OP care in both OBG and radiotherapy departments, was contributed by the salaries of the staff. It was followed by capital cost (21% and 16%) respectively. Further, it was seen that, the cost of equipment (35%) constituted the major portion of the total cost incurred on 3D-CRT. For brachytherapy, salaries constituted was the single largest component (45%) of the total cost.

*Out of Pocket expenditure*

*Sample characteristics*

Among the patients recruited, 52% (129/248) were aged between 45-60 years, 46% (115/248) were illiterate, 72% (177/248) belonged to Hindu religion, 62% (154/248) resided in rural areas, and 70% (174/248) reported not having any health insurance (Supplementary material; Table S1). At the time of diagnosis, around 65%, and 25% of the patients were in either stage I/II or, stage III/IV respectively. About 10% of the patients have unknown stage at time of reporting to the study hospital . 

*Out-of-pocket expenditure (OOPE)*

Stage-specific mean OOPE incurred by a cervical cancer patient varied from INR 27,886 (24,782-30,990) for a patient in stage III to INR 46,382 (39,555-53,210) for a patient treated in stage I as shown in Table 3. In terms of specific treatment procedure, OOPE was highest for surgery (INR 23,453 (95% CI: 17,594-29,314)), followed by radiotherapy (INR 12,822; 95% CI: 11,696-13,948), brachytherapy (INR 5,583; 95% CI: 4,919-6,246) and chemotherapy (INR 4,042; 95% CI: 3,436-4,648) as shown in table 3. Further, a mean OOPE of INR 15,937 (11,341-20,533) was incurred by the patients before coming to the study hospital. This OOPE was significantly associated with higher education, stage of cancer and type of the treatment mortality (Table 2).

*Financial Risk Protection*

Among the recruited patients, 62% (n=155) suffered from CHE at the 40% threshold. On changing the threshold to 20%, 30% and 50%, the prevalence of CHE changed to 86%, 71% and 52% respectively. Logistic regression at 40% threshold showed that the odds of CHE were significantly higher for patients in lower-income quintile patients (OR: 25.39, p-value: <0.001), as compared to the highest income quintile (Supplementary material; Table S2). 

About 30% of the patients (n=75) reported having undertaken borrowing or selling off assets to cope up with the expenditure incurred on the cervical cancer treatment. The odds of distress financing was highest among the poorest income quintile (OR: 15.37, p-value: 0.001) (Supplementary material; Table S3).

*Health Benefit Package (HBP) Cost*

The HBP cost for brachytherapy, surgery and radiotherapy is INR 45,364, 56,538 and 60,422 respectively (Table 4). 

**Table 1:** Health System Cost for Services Provided for Cervical Cancer Treatment at a Tertiary Level Public Sector Hospital

Department	Service	Unit	Unit Cost (INR)
Obstetrics & Gynaecology	Outpatient consultation	per patient visit	324
	Inpatient care	per bed day	2,742
	Radical Hysterectomy	per patient	19,494
Radiation Oncology	Outpatient consultation	per patient visit	547
	3-dimensional conformal radiotherapy (3D-CRT)	per patient	41,388
	Brachytherapy	per patient	33,569
	Diagnostics	per patient	3,052

**Table 2:** Out of Pocket Expenditure Incurred During Treatment of Cervical Cancer

Variable	Category	Mean OOP (95% CI)	p-value
Age	Less than 45 years	38,957 (32,560-45,353)	0.141
	45-60 years	34,583 (31,446-37,721)	
	60 years and above	31,865 (27,226-36,504)	
Education	Illiterate	28,381 (25,963-30,798)	<0.001
	Primary	40,452 (33,301-47,603)	
	Secondary	37,935 (32,561-43,309)	
	Senior Secondary & above	45,110 (36,570-53,649)	
Locality	Urban	35,949 (31,680-40,218)	0.517
	Rural	34,274 (31,245-37,303)	
Insurance	Yes	29,903 (25,597-34,209)	0.009
	No	37,038 (34,069-40,006)	
Income Quintiles	Poorest	34,881 (28,549-41,212)	0.364
	Poor	32,390 (28,189-36,591)	
	Middle	32,197 (26,855-37,538)	
	Rich	35,381 (29,132-41,630)	
	Richest	39,489 (34,338-44,639)	
Stage of cervical cancer	Stage 1	46,382 (39,555-53,210)	
	Stage 2	33,955 (30,570-37,340)	<0.001
	Stage 3	27,886 (24,782-30,990)	
	Stage 4	32,739 (6,359-71,838)	
	Unknown stage	39,238 (27,543-50,934)	
Treatment	Radiotherapy alone	26,037 (20,789-31,284)	<0.001
modality	Radiotherapy along with Brachytherapy	32,813 (25,075-40,552)	
	Radiotherapy along with Chemotherapy	25,270 (20,646-29,895)	
	Radiotherapy along with brachytherapy and chemotherapy	36,615 (33,516-39,714)	
	Surgery followed by other treatment modalities	43,541 (34,518-52,563)	
Total out of pocket expenditure		35,741 (32,969-38,513)	

**Table 3:** Treatment Specific Direct & Non-Direct Medical Out of Pocket Expenditure for Cervical Cancer Treatment

Treatment procedure	Direct medical expenditure in INR (95% CI)	Direct non-medical expenditure in INR (95% CI)	Total expenditure in INR (95% CI)
Before coming to study hospital	10,786 (6,565 -15,007)	1,830 (973-2,687)	15,937 (11,341-20,533)
Pre-radiotherapy*	5,830 (5,185-6,474)	5,124 (4,135-6,114)	10,949 (9,573-12,325)
Radiotherapy	3,547 (3,245-3,848)	9,275 (8,256-10,294)	12,822 (11,696-13,948)
Brachytherapy	3,755 (3,392-4,118)	1,828 (1,361-2,294)	5,583 (4,919-6,246)
Chemotherapy	3,210 (2,720-3,699)	832 (652-1,013)	4,042 (3,436-4,648)
Surgery	21,850 (16,205-27,494)	1,603 (1,113-2,094)	23,453 (17,594-29,314)

*Pre-radiotherapy expenditure includes expenditure incurred during the preliminary investigations in the outpatient clinic on the Obstetrics and Gynaecology department.

**Table 4:** Health Benefit Package (HBP) Rates for Different Treatment Modalities for Cervical Cancer Across Various Publicly Financed Health Insurance Schemes

Treatment Modality	Package rates under various Insurance schemes in INR					
	RSBY^*^	AB PM-JAY^$^	CMCHIS^#^	MJPJAY^@^	Aarogyasri^+^	Present study
3-Dimensional conformal radiotherapy	75,000	50,000	75,000	75,000	75,000	60,422
Brachytherapy (Interstitial)	15,000	30,000	15,000	15,000	15,000	45,364
Radical hysterectomy	15,000	20,000	30,000	30,000	30,000	56,538

^*^, Rashtriya Swasthiya Bima Yojana; ^$^, Ayushman Bharat Pradhan Mantri-Jan Arogya Yogana; ^#^, Chief Minister Comprehensive Health Insurance Scheme, Tamil Nadu; ^@^, Mahatma Jyotiba Phule Jan Arogya Yojana, Maharashtra; ^+^, Aarogyasri Health Care Trust, Telangana

## Discussion

The high disease burden and rising cost of cancer treatment imposes a huge financial burden both on the health systems and households. In India, only 12% and 13% of the urban and rural population have health insurance coverage respectively, and around 60% of the health care expenditure is paid out-of-pocket by households. Therefore, diagnosis of cancer becomes devastating news for the household due to financial and psychological hardships (National Health Systems Resource Centre 2015; National Sample Survey Organization, 2015). Evidence in impact of publicly financed health insurance schemes suggest that there has been no decline in the OOPE payments (Prinja et al., 2017). Moreover, as India has launched the world’s largest tax-funded health insurance scheme Ayushman Bharat-Pradhan Mantri Jan Arogya Yojana (AB - PMJAY) (Angell et al., 2019), the need of cost data for various treatment regimens available for cancer treatment gains considerable importance in designing appropriate provider payment rates that could adequate financial risk protection. The present study was designed to estimate the cost of HBPs for the treatment of cervical cancer in India.

Present study comprehensively estimated the total cost of cervical cancer treatment considering both the health system cost and OOPE. In context of public sector hospitals in India, while treatment is subsidized, patients still have to pay OOPE for drugs, consumables and diagnostics. Thus, it becomes necessary to estimate both the health system cost and OOPE while estimating the total cost of treatment. There is only a single study which comprehensively assessed the cost of head and neck cancer treatment from societal perspective (Chauhan et al., 2018). Other studies were either focussed on health system cost or OOPE (Chatterjee et al 2013; Chauhan et al., 2013; Mahal et al., 2013; Rajpal et al 2018). 

*Comparison of OOPE and financial risk protection*

A systematic review focusing on low and middle-income countries (LMICs) reported that non-communicable diseases (NCDs) affected households spend 5% and 59% of either the household income or consumption expenditure or non-food consumption expenditure as OOPE (Kankeu et al., 2013). Another review from the same region reported CHE due to NCDs in range of 0-34% (Alam et al., 2014). Further, a study focusing on cancer conducted across 8 countries of the south-east region (SEAR) stated the prevalence of CHE of 48% as compared to 64% in the present study (Kimman et al., 2015). The findings of these studies are difficult to compare due to variation in the methodology for measuring catastrophic spending. Firstly, the threshold for CHE is either relative to total household expenditure or ‘non-food expenditure’ in different studies. Secondly, the level of the threshold varied from 10% to 40%. The study hospital is a tertiary public hospital which is a referral site for patients from about 6 states. Many patients approach with advance stages of cervical cancer leading to higher CHE. Further, prevalence CHE is affected by lack of screening, late detection, inadequate referral mechanism and treatment modality used. 

The SEAR study reported higher odds of incurring CHE in lower-income quartiles and without health insurance. We also found a higher vulnerability of poor for CHE. However, we found no protection from CHE as a result of insurance. A previous review of health insurance schemes in India also supports the findings of our study citing lack of protective effect of insurance on catastrophic spending. This could be due to the design features and purchasing mechanisms under current PFHIs.

On comparison of HBP rates under various publicly financed health insurance schemes, the HBP rates for 3-dimensional conformal radiotherapy (3D-CRT) varies from INR 75,000-90,000 whereas in the present study HBP cost is INR 60,422. (Table 4) Thus, there is a need for refining HBP rates of PFHIs based on scientific costing studies.

Further, about 60% of recruited patients were from rural areas, who have to incur additional expenses in the form of travelling and boarding/lodging. This is reflected through a high proportion of non-direct OOPE ranging from 72% to 33% while getting treatment with radiotherapy to brachytherapy respectively. It was observed that the OOPE on treatment was highest in stage I, this is possibly due to requirement of more aggressive treatment in this stage including surgery. Thus, there is a need for developing an adequate network of facilities so that patients do not have to travel far from home for getting cancer treatment. The National cancer control programme also recommends the strengthening of district hospitals to provide radiotherapy.

*Methodological issues*

Mixed (top-down and bottom-up) micro-costing methods were followed for estimating the health system cost. Due lack of disaggregated data and patient records in physical form, a pure bottom-up costing approach was not possible. 

Standard Cost of Illness approach was used for estimating the OOPE. Further, among the total recruited patients interviewed for OOPE, around 1/4th were interviewed prospectively and remaining were interviewed retrospectively following up to 6 months of the treatment. The national sample survey of India recommends a reference period of the last 365 days for assessing the expenditure incurred in rare events like hospitalization. Cancer treatment in the form of surgery or radiotherapy/brachytherapy given either alone or in combination usually spans over the duration of 3-4 weeks. Hence, a recall period of up to 6 months was considered appropriate. Moreover, there was no significant difference in the average OOPE among those patients recruited prospectively or retrospectively, suggesting absence of any systematic recall bias. 

For some patients undergoing chemotherapy, the drugs were provided free by the hospital (poor free) or government-sponsored schemes like Mukh Mantri Punjab Cancer Raahat Kosh Scheme (MMPCRKS). The number of such patients could not be captured during the data collection. Hence, the cost was neither covered in health systems cost nor OOPE. Therefore, the cost of chemotherapy in the present study is underreported.

In conclusion, high OOPE incurred on cancer treatment results in a lack of adequate financial risk protection. The financial hardship is particularly high for the poorest section of society, which raises important equity issues. Since majority of the OOPE in public sector is for drugs and diagnostics, this calls for strengthening the capacity of the existing public health sector for better availability of drugs and diagnostic services such that patients are not forced to spend out-of-pocket. Secondly, high rates of catastrophic health expenditure on account of cancer treatment imply that there is a need to enhance coverage of risk pooling mechanisms for reducing reliance on OOP payments. Although PFHIs provide coverage for cancer treatment, there is a need to adequately revise the provider payment rates, such that there is no co-payment from patients. The findings of our study indicate that there is a need to suitably revise the provider payment rates. Finally, besides provision of free treatment, there is a need to focus on prevention interventions such as screening for cervical cancer and vaccination against human papilloma virus, which have been shown to be cost effective in India (Prinja et al., 2017C). 

## Funding Statement

The study was funded by the Department of Health Research, Ministry of Health and Family Welfare, Government of India.
